# Directed evolution of α-ketoisovalerate decarboxylase for improved isobutanol and 3-methyl-1-butanol production in cyanobacteria

**DOI:** 10.1186/s13068-025-02687-6

**Published:** 2025-07-31

**Authors:** Hao Xie, Afshan Begum, Laura H. Gunn, Peter Lindblad

**Affiliations:** 1 Microbial Chemistry, Department of Chemistry-Ångström Laboratory, Uppsala University, Uppsala, Sweden; 2 College of Bioengineering, Sichuan University of Science & Engineering, Yibin, Sichuan China; 3 Department of Cell and Molecular Biology, Uppsala University, Uppsala, Sweden; 4 Plant Biology Section, Cornell University, Ithaca, NY USA

**Keywords:** Cyanobacteria, *Synechocystis*, Isobutanol, 3-Methyl-1-butanol, Directed evolution, *α*-Ketoisovalerate decarboxylase

## Abstract

**Background:**

Cyanobacteria are promising platforms for metabolic engineering to convert carbon dioxide into valuable fuels and chemicals, addressing both energy demands and global climate change. Among various fuels and chemicals, isobutanol (IB) and 3-methyl-1-butanol (3M1B) have gained increasing attention due to their superior physical properties, such as high energy density, low water solubility, and low hygroscopicity. Heterologously expressing *α*-ketoisovalerate decarboxylase (Kivd^S286T^) in the unicellular cyanobacterium *Synechocystis* sp. PCC 6803 (*Synechocystis*) enables microbial production of IB and 3M1B through the 2-keto acid pathway, with Kivd^S286T^ identified as a key bottleneck limiting production efficiency.

**Results:**

To address this limitation, a high-throughput screening method based on the consumption of the substrate 2-ketoisovalerate was successfully established. This screen was coupled with random mutagenesis, via error-prone PCR, of Kivd^S286T^. Out of the 1600 variants, 1B12, featuring dual substitutions K419E and T186S, exhibited a 55% increase in IB production and a 50% increase in 3M1B production in *Synechocystis* on the fourth day of cultivation. The crystal structure of Kivd^S286T^ was determined as a tetramer with a resolution of 2.8 Å to provide a framework for analyzing the structural basis for the enhanced butanol production conferred by the K419E and T186S substitutions.

**Conclusions:**

A novel Kivd variant, 1B12, was successfully generated via directed evolution, with enhanced catalytic activity for microbial IB and 3M1B biosynthesis. To our knowledge, this study represents the first successful application of directed evolution on the rate-limiting enzyme of a specific metabolic pathway to enhance biochemical production in cyanobacteria.

**Supplementary Information:**

The online version contains supplementary material available at 10.1186/s13068-025-02687-6.

## Introduction

Isobutanol (IB) is an alcohol compound widely used as a raw material across various industrial sectors to produce materials, such as coatings, plastics, rubber, pesticide, and pharmaceuticals [1]. Recent developments have highlighted IB’s potential as a new-generation biofuel due to its favorable properties, such as lower hygroscopicity, lower volatility, and higher energy density compared to ethanol [1, 2]. Classified as an advanced biofuel, IB shares fuel characteristics closely resembling those of gasoline, making it compatible with existing storage and transportation infrastructure. Consequently, IB can directly replace conventional fuels or serve as a fuel additive. Currently, IB is synthesized through chemical processes in industry, relying on petrochemical-derived feedstocks and energy-intensive methods [1, 3–5]. In light of increasing energy demands and growing environmental concerns, there is an urgent need to shift IB production from chemical processes to renewable processes. Leveraging advances in biotechnology, microbial production of IB has been successfully achieved in various heterotrophic micro-organisms using α-ketoisovalerate decarboxylase (Kivd) from *Lactococcus lactis* [6–8]. Furthermore, carbon–neutral IB production has been achieved in photoautotrophic micro-organisms, particularly cyanobacteria, which utilize CO_2_ as carbon source and sunlight as energy source [9–11]. Recently, both CRISPR inteference and CRISPR activation systems have been successfully implemented in IB-producing cyanobacteria, demonstrating their effectiveness as tools for enhancing IB production [12, 13].

**Fig. 1:**
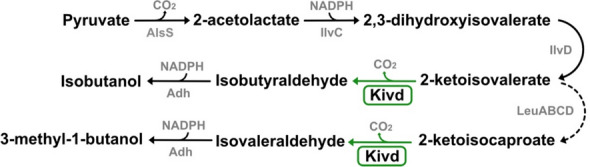
Biosynthetic pathway for isobutanol and 3-methyl-1-butanol production in engineered *Synechocystis* sp. PCC 6803 strain. The enzymes colored in gray are native to *Synechocystis*, while the enzyme colored in black is sourced from *Lactococcus lactis*. The native reactions are indicated by black arrows; the heterologous reaction is indicated by green arrow; and the multiple-step reaction is indicated by a dashed arrow. Abbreviations for enzymes: AlsS, acetolactate synthase; IlvC, acetohydroxy-acid isomeroreductase; IlvD, dihydroxy-acid dehydratase; Kivd, *α*-ketoisovalerate decarboxylase (*L. lactis*); Adh, alcohol dehydrogenase; LeuA, 2-isopropylmalate synthase; LeuCD, 3-isopropylmalate dehydratase; LeuB, 3-isopropylmalate dehydrogenase

Heterologous expression of Kivd in the unicellular cyanobacterium *Synechocystis* sp. PCC 6803 (*Synechocystis*) enables IB production, with 3-methyl-1-butanol (3M1B) produced as a by-product (Fig. 1) [10]. The IB biosynthesis pathway, known as the 2-keto acid pathway, involves a cascade of five enzymatic reactions. Kivd, the key enzyme in this pathway, catalyzes the conversion of 2-ketoisovalerate into isobutyraldehyde, which has been identified as a bottleneck in IB production [10]. Kivd, a thiamine diphosphate (ThDP)-dependent 2-keto acid decarboxylase, belongs to the pyruvate decarboxylase family and exhibits broad substrate specificity [14]. The enzyme demonstrates the highest affinity for 2-ketoisovalerate, an intermediate in the biosynthesis of the branched-chain amino acid valine [14]. The catalytic mechanism of Kivd involves the following steps: coordinated by a Mg^2+^ ion, ThDP in the active site undergoes deprotonation, forming the active ylide-form with the assistance of a glutamate residue; upon the entry of substrate, the active-form ThDP reacts with the carbonyl group of the substrate forming a covalent intermediate; subsequently, decarboxylation of the intermediate occurs, leading to the release of CO_2_ and the generation of an active aldehyde; finally, the enamine intermediate is protonated and the corresponding aldehyde is released [15–17].

To address the bottleneck in the 2-keto acid pathway and enhance IB production, rational design strategies were employed to modify the Kivd enzyme. S286T or V461I substitution conferred more than a threefold increase in IB production in *Synechocystis* [18]. Further enhancement of IB production was achieved by manipulating *kivd*^*S286T*^ copy numbers and optimizing expression units at both the transcriptional and translational levels [19, 20]. However, elevating the overall cellular expression of heterologous enzymes may impose metabolic burdens on cellular growth and productivity [21].

Beyond rational design and optimizing expression, directed evolution of rate-limiting enzyme(s) is an additional strategy to enhance catalytic activity and overcome bottlenecks in IB production. In rational design, specific amino acid mutations are introduced based on knowledge of an enzyme, e.g., protein crystal structure, position of active site, and utilization of cofactors. However, accurately predicting the positive effects of specific amino acid mutations on protein performance remains challenging [22]. Directed evolution offers distinct advantages over rational design, bypassing the need for precise mutation identification and simulating natural evolution in a laboratory setting within a much shorter timeframe [23]. The process involves selecting a starting sequence, constructing a mutant library, and identifying variants with improved activity [24]. While constructing an efficient high-throughput screening method for identifying desired variants can be challenging, directed evolution has been effective in producing enzyme variants with enhanced catalytic activity, stability, and selectivity [25, 26]. This approach has been widely applied to the bioproduction of fuels, chemicals, and pharmaceutical intermediates [27, 28]. Rational design of Kivd has been employed to selectively produce desired chemicals [18, 29–31]. Recently, directed evolution was used to generate thermostable Kivd variants, although these thermostable variants have not yet been tested in lignocellulosic thermophiles for keto acid derived alcohol production [32].

In this study, we developed and implemented a platform for generating and selecting Kivd^S286T^ variants with enhanced catalytic activity. A high-throughput screening method based on absorbance changes at 313 nm, allowing direct measurement of the consumption of the substrate 2-ketoisovalerate, was established and validated. This method was then used to screen four Kivd^S286T^-derived libraries, consisting of in total 1600 independent variants constructed via error-prone PCR. The top eight best-performing Kivd^S286T^ variants were subsequently transformed into wild-type *Synechocystis* to evaluate IB and 3M1B production. Among these, variant 1B12, which possesses dual mutations (T186S and K419E), demonstrated significant enhancements in IB and 3M1B production. Novel Kivd^S286T^ variants with either the T186S or K419E mutation individually were generated and characterized. Furthermore, we solved the crystal structure of Kivd^S286T^, providing insights into the improved performance of selected Kivd^S286T^ variants.

## Materials and methods

### Bacterial strains and growth conditions

The *Escherichia coli* (*E. coli*) strain DH5α-Z1 (Invitrogen) was employed for cloning purposes, while *E. coli* strain HB101, containing the plasmid pRL443-AmpR, served as the helper strain for conjugation. For protein expression, library construction, and high-throughput screening, *E. coli* strain BL21 (DE3) (Invitrogen) was employed. All *E. coli* cultures were maintained at 37 °C in lysogeny broth (LB) medium (Sigma-Aldrich), supplemented with appropriate antibiotics (50 μg mL^−1^ kanamycin, 100 μg mL^−1^ ampicillin), unless otherwise specified. Throughout the study, the glucose-tolerant *Synechocystis* sp. PCC 6803 (*Synechocystis*) was used, with seed cultures maintained at 30 °C under 30 μmol photons m^−2^ s^−1^ in BG11 medium [33].

### Plasmid construction and library construction

The plasmids constructed and used in this study are detailed in Table S1. Plasmid pHX_EVC was constructed by circularizing a DNA fragment amplified from plasmid pHUE [34] using primers pHX_F and pHX_R (Table S2). Plasmid pHX_ST was assembled by ligating the vector backbone and the *kivd*^*S286T*^ gene fragment via HiFi DNA Assembly (New England Biolabs). The vector backbone was amplified from plasmid pHUE using primers pHUE_F and pHUE_R, while the *kivd*^*S286T*^ gene fragment was amplified from pUC57_ST with primers kivd_F and kivd_R. The GeneMorph II random Mutagenesis Kit (Agilent Technologies) was employed for library construction. Error-prone PCR (EP-PCR) was performed on *kivd*^*S286T*^ using Mutazyme II DNA polymerase, with pUC57_ST as the template and kivd_F and kivd_R as the amplification primers. To achieve 1–4 mutations per *kivd*^*S286T*^ gene fragment, 300 ng of the template was subjected to 25 cycles of PCR. Vector backbone amplification from plasmid pHUE was carried out using Phusion Polymerase (Thermo Scientific) with pHUE_F and pHUE_R as amplification primers. PCR templates were removed by DpnI (Thermo Scientific) digestion at 37 °C for 1 h. The *kivd*^*S286T*^ random mutagenesis library was then ligated with the vector backbone using HiFi DNA Assembly. The ligation mixture was transformed into *E. coli* DH5α-Z1 cells for library quality assessment or *E. coli* BL21 (DE3) cells for high-throughput screening. To evaluate the mutation rate, 8–12 independent transformants were randomly selected and sequenced. The pEEK2 series plasmids were constructed by inserting *kivd*^*S286T*^ variants into the pEEK2 vector [10]. The *kivd*^*S286T*^ variants were amplified from the corresponding pHX series plasmids (Table S1) using primers kivd_BglII_F and kivd_SpeI_R. Plasmid pHUE_ST was constructed by ligating the *kivd*^*S286T*^ fragment with the pHUE linear fragment. The *kivd*^*S286T*^ fragment was amplified from pUC57_ST and digested with FastDigest enzymes XbaI and EcoRI (Thermo Scientific), while the pHUE plasmid was digested with XbaI, EcoRI, and FastAP (Thermo Scientific). All DNA fragments, except those for the *kivd*^*S286T*^ random mutagenesis library, were amplified using Phusion polymerase. All primers were purchased from IDT technologies and are detailed in Table S2.

### Cell cultivation and cell lysis for high-throughput screening

Single cell colony was picked and cultured in 96-deep-well (DW) plate (VWR) containing 200 μL terrific broth (TB) medium supplemented with 3% (v/v) glycerol and 100 μg mL^−1^ ampicillin. The plates were sealed with Adhesive Gas Permeable Seals (Thermo Scientific) and incubated at 37 °C with a rotation speed of 190 rpm until the OD_595_ reached between 0.4 and 0.5. Subsequently, a copy of the cultured colonies was transferred to a new 96-DW plate filled with 200 μL TB medium supplemented with 10% (v/v) glycerol and 100 μg mL^−1^ ampicillin. This plate, designated as the storage copy, was incubated overnight, sealed with Nunc Aluminum Seal Tape (Thermo Scientific), and stored at − 80 °C. Following the preparation of the storage copy, 10 μL of 21 mM IPTG were added to the original plate to achieve a final concentration of 1 mM IPTG to induce protein expression. The induction was carried out for 15 h at 25 °C with shaking speed at 160 rpm, followed by OD_595_ measurement.

For cell lysis, 80 μL of 3X BugBuster Protein Extraction Reagent (Millipore) were added to each cell culture. The mixtures were incubated at room temperature on a shaker at 80 rpm for 20 min. Insoluble cell debris were subsequently removed by centrifugation at 3700 xg for 20 min, and the resulting soluble fraction was collected for the activity assay.

### KIV (KIC) absorbance-based Kivd activity assay for high-throughput screening

#### Initial conception of KIV and KIC absorbance-based Kivd activity assay

Both 2-ketoisovalerate (KIV) and 2-ketoisocaproate (KIC) exhibit an absorbance peak at 313 nm, as determined through spectral analysis. This suggests that changes in absorbance at 313 nm can serve as indicators of KIV and KIC consumption. To validate this, a series of aqueous solutions containing KIV or KIC at concentrations of 0 mM, 5 mM, 10 mM, 20 mM, 40 mM, or 60 mM were prepared. The absorbance at 313 nm (A_313_) was then measured and plotted against the corresponding KIV or KIC concentrations.

#### Assay development and validation with E. coli crude protein soluble fractions

Two *E. coli* strains, HXEC_EVC and HXEC_ST, were employed for assay development and validation (Table 1). The cells were cultured and lysed as described in Sect. "Cell cultivation and cell lysis for high-throughput screening". For Kivd activity assay targeting KIV and KIC, 30 μL of soluble protein supernatant were added to each well of two separate 96-well plates (SARSTEDT). A 170 μL enzyme master mix was then added to each well, achieving final concentrations of 5 mM MgCl_2_, 1.5 mM thiamine diphosphate (ThDP), and 60 mM KIV or KIC in a 50 mM sodium phosphate buffer at pH 6.5. The A_313_ was recorded as the baseline (start-point) prior to incubation at 30 °C. After incubation periods of 5 h, 20 h, 25 h, or 45 h, the A_313_ was measured again to determine the end-point reading of enzyme assay. Enzyme activity was subsequently calculated using two methods: ΔA_313_ (start-point A_313_ − end-point A_313_) and ΔA_313_ OD_595_^−1^.

**Table 1:** List of strains used

Strains	Relevant genotypes^a^	References
*Synechocystis* sp. PCC 6803 strains		
6803 WT	wild-type *Synechocystis* sp. PCC 6803	[36]
HX_EVC	pEEK_P*trc*_RBS_T_KanR	This study
HX_ST	pEEK2_P*trc*BCD_***Strep-ST***_T_KanR	This study
HX_1B12	pEEK2_P*trc*BCD_***Strep-1B12***_T_KanR	This study
HX_1C9	pEEK2_P*trc*BCD_***Strep-1C9***_T_KanR	This study
HX_2A11	pEEK2_P*trc*BCD_***Strep-2A11***_T_KanR	This study
HX_3C4	pEEK2_P*trc*BCD_***Strep-3C4***_T_KanR	This study
HX_4B7	pEEK2_P*trc*BCD_***Strep-4B7***_T_KanR	This study
HX_4H5	pEEK2_P*trc*BCD_***Strep-4H5***_T_KanR	This study
HX_5F1	pEEK2_P*trc*BCD_***Strep-5F1***_T_KanR	This study
HX_14G8	pEEK2_P*trc*BCD_***Strep-14G8***_T_KanR	This study
HX_T186S	pEEK2_P*trc*BCD_***Strep-T186S***_T_KanR	This study
HX_K419E	pEEK2_P*trc*BCD_***Strep-K419E***_T_KanR	This study
*E. coli* strains		
HXEC_EVC	pHX_T7_RBS_T_AmpR	This study
HXEC_ST	pHX_T7_RBS_***ST***_T_AmpR	This study
HXEC_1B12	pHX_T7_RBS_***1B12***_T_AmpR	This study
HXEC_1C9	pHX_T7_RBS_***1C9***_T_AmpR	This study
HXEC_2A11	pHX_T7_RBS_***2A11***_T_AmpR	This study
HXEC_3C4	pHX_T7_RBS_***3C4***_T_AmpR	This study
HXEC_4B7	pHX_T7_RBS_***4B7***_T_AmpR	This study
HXEC_4H5	pHX_T7_RBS_***4H5***_T_AmpR	This study
HXEC_5F1	pHX_T7_RBS_***5F1***_T_AmpR	This study
HXEC_14G8	pHX_T7_RBS_***14G8***_T_AmpR	This study
HXEC_H6-Ub_ST	pHUE_T7_RBS_H6-Ub_***kivd***^***S286T***^_T_AmpR	This study

^a^KmR, kanamycin resistance cassette; AmpR, ampicillin resistance cassette; T, Terminator BBa_B0015. Expressed genes in bold

#### Assay application in high-throughput screening

Cells were cultured and lysed as outlined in Sect. "Cell cultivation and cell lysis for high-throughput screening". For KIV absorbance-based Kivd assay, 30 μL of soluble protein supernatant was added to a 96-well plate (SARSTEDT). Subsequently, 170 μL of enzyme master mix was added to each well, achieving final concentrations of 5 mM MgCl_2_, 1.5 mM ThDP, and 60 mM KIV in 50 mM sodium phosphate buffer at pH 6.5. The A_313_ was recorded as the baseline (start-point) prior to incubation at 30 °C. After an incubation period of 2 h, the A_313_ was measured again to obtain the end-point reading. Enzyme activity was calculated as ΔA_313_ (start-point A_313_ − end-point A_313_).

### Cyanobacteria strain construction

The pEEK* and pEEK2 series plasmids containing *kivd*^*S286T*^ variants were individually introduced into the wild-type *Synechocystis* strain via three parental mating [35], as previously described [19]. Single colonies were then streaked onto new BG11 agar plates supplemented with 50 μg mL^−1^ kanamycin. Positive colonies were verified by colony PCR using the primer pairs VF2/VR for the pEEK* plasmid and VF2/kivd_SR for the pEEK2 series plasmids. Three positive colonies from each conjugation event were retained for further analysis. All cyanobacterial strains used and generated in this study are listed in Table 1.

### Synechocystis cultivation condition

*Synechocystis* seed cultures were grown at 30 °C in BG11 medium supplemented with 50 μg mL^−1^ kanamycin, using 100 mL Erlenmeyer flasks (VWR), under a light intensity of 30 μmol photons m^−2^ s^−1^. These seed cultures were then used to inoculate experimental cultures in BioLite 25 cm^2^ plug-sealed tissue culture flasks (Thermo Scientific), with a culture volume of 25 mL and an initial OD_750_ of 0.1. The medium used for experimental culture consisted of BG11, supplemented with 50 mM NaHCO_3_ and 50 μg mL^−1^ kanamycin. All experimental cultures were conducted in biological triplicates and shaken horizontally at 120 rpm, under a light intensity of 50 μmol photons m^−2^ s^−1^ at 30 °C. Cell growth was assessed by measuring optical density at 750 nm, as previously described [19]. Every second day, 2 mL of culture were sampled for OD_750_ and product measurements, and 2 mL of fresh medium, supplemented with 500 mM NaHCO_3_ and 50 μg mL^−1^ kanamycin, were added back. The cultivation period lasted for 8 days, with OD_750_ measured daily and product concentrations measured every second day.

### Product analysis

Isobutanol (IB) and 3-methyl-1-butanol (3M1B) were extracted from culture supernatant every second day using dichloromethane (DCM), following established protocols [19]. Quantification of IB and 3M1B was carried out using a PerkinElmer GC 580 system, equipped with a flame ionization detector and an Elite-WAX Polyethylene Glycol Series Capillary column, 30 m × 0.25 mm × 0.25 μm (PerkinElmer). The detailed analytical program is described in [10, 19].

### Crude protein extraction and SDS–PAGE/Western-immunoblot

Crude protein extraction and SDS–PAGE/Western-immunoblot were conducted following previously described methods [19]. Five micrograms of soluble crude proteins were loaded for protein expression analysis.

### Protein expression in E. coli BL2 (DE3) strain

The plasmid pHUE_ST was used to express Kivd^S286T^ in *E. coli* BL21 (DE3) cells (Tables 1 and S1). The *E. coli* cells were cultured in 2 L LB medium supplemented with 100 μg mL^−1^ ampicillin at 37 °C. Protein expression was induced with isopropyl-β-D-thiogalactopyranoside (IPTG) at a final concentration of 1 mM when the cells reached mid-log phase, followed by incubation at 30 °C for 4 h. H6-Usp2cc was expressed and purified according to previously established protocols [34].

### Protein purification

The purification of Kivd^S286T^ followed a multistep protocol involving immobilized metal affinity chromatography (IMAC), H6–Ub tag cleavage and subsequent removal, followed by size-exclusion chromatography. All columns employed in each purification step were connected to an NGC chromatography system (Bio-Rad).

Cells were harvested by centrifugation at 8000 xg for 15 min at 4 °C and then resuspended in cell lysis buffer (20 mM sodium phosphate, pH 7.4, 500 mM NaCl, 10 mM imidazole, 5 mM MgCl_2_, protease inhibitor tablets, 1 μg mL^−1^ DNase I). Following resuspension, the cells were lysed by sonication treatment (50% amplitude, 5 s on/10 s off, 6 min). Soluble proteins were obtained by centrifugation (Beckman JL-25.50) at 48,000 xg for 20 min at 4 °C. The obtained protein supernatant was filtered through a 0.2 µm filter and loaded onto a HisTrap HP His tag protein purification column (Cytiva), which had been pre-equilibrated with lysis buffer. Protein elution was carried out using an elution buffer (20 mM sodium phosphate, pH 7.4, 500 mM NaCl, 500 mM imidazole). Peak fractions were analyzed by SDS–PAGE (Fig. S1), and fractions containing protein bands were pooled. The H6–Ub tag was then cleaved using H6–Usp2cc, following previously described protocols [37].

The cleaved protein was filtered through a 0.2 µm filter and subjected to size-exclusion chromatography using a HiLoad 26/60 Superdex 200 column (Cytiva). Peak fractions were analyzed by SDS–PAGE (Fig. S1), and fractions within the peak area were pooled. To remove H6–Ub tag, uncleaved protein, and H6–Usp2cc, a final purification step was performed using a HisTrap HP His tag protein purification column. The purified protein was then stored in size-exclusion Superdex buffer (20 mM sodium phosphate, pH 6.5, 50 mM NaCl, 2.5 mM MgSO_4_, 0.1 mM ThDP), concentrated, and kept at − 80 °C for long-term storage.

### Protein crystallization of Kivd^S286T^

Protein crystallization was performed using the hanging-drop vapor-diffusion method. Initial crystallization conditions were determined with the pHClear Suite (Molecular Dimension) commercial crystallization screen. Optimization was then conducted in 24-well hanging-drop plates, with 1 mL reservoir solution containing 16–17.8% (w/v) PEG6000 or PEG3350 in 0.1 M MES buffer at pH 6.0. The crystallization drops were prepared by mixing 2 μL of protein solution (protein concentration: 10 mg mL^−1^ for Kivd^S286T^) with 2 μL of reservoir solution, and allowed to equilibrate over a 1 mL reservoir solution in a Linbro plate (Hampton Research) at 20 °C for 1–3 weeks. Prior to data collection, the crystals were transferred to a cryoprotectant solution containing 20% v/v ethylene glycol in the mother liquor. The crystals were then harvested and flash-cooled in liquid nitrogen.

### Data collection and structure determination of Kivd^S286T^

Diffraction data were collected on beamline I03 of the Diamond Light Source, Oxfordshire, UK. Data collection statistics are provided in Table 2. The data set collected from a single crystal was integrated with XDS [38] and further processed with POINTLESS, and AIMLESS as implemented in the CCP4i2 graphical user interface [39], which revealed that the crystals belonged to the space group C2.

**Table 2:** Data collection and refinement statistics for Kivd^S286T^ Values in parentheses are for the outer shell

Data collection	
Beamline	Diamond
Wavelength (Å)	0.9762
Space group	C2
Unit cell parameters (Å)	*a* = 218.5, *b* = 81.7, *c* = 124.9
Resolution limits (Å)	40.0–2.79 (2.81–2.79)
Total no. of reflections	463,917
No. of unique reflections	66,225
Multiplicity	7.0 (6.6)
Completeness (%)	99.7 (99.43)
R_merge_	0.110 (0.026)
R_meas_	0.12 (0.029)
*I*/σ (*I*)	12.0 (0.9)
CC_1/2_	0.999 (0.30)

Refinement	
Resolution Range (Å)	40.0–2.79
R_factor_ (%)	18.40
R_free_ (%)	23.80
No. of ligands	2
No. of residues	2087
No. of water molecules	30
No. of Mg^2+^	2
Average *B* values (Å^2^)	48.69
r.m.s. deviations from ideal values	
Bond distances (Å)	0.009
Bond angles (^o^)	1.74
Ramachandran plot	
Residues favored regions (%)	96.5
Residues allowed regions (%)	3.2
Ramachandran outliers (%)	0.34

The X-ray model of alpha-ketoisovalerate decarboxylase (KivD) (PDB entry 6VGS) and X-ray intensity data from 40.0 to 2.8 Å resolution were used in molecular replacement searches with the program Molrep [40, 41] to recover the phase information. The Matthews coefficient for Kivd^S286T^ was calculated to be 2.21 Å^3^ Da^−1^ [42]. This suggests the presence of four molecules (two dimers) of each Kivd^S286T^ in one asymmetric unit. The models were refined against all diffraction data using RAFMAC5 [43]. The model was edited manually using COOT [44]. Further iterative rounds of refinement were carried out in RAFMAC5 with the CCP4-cloud server [45]. Five percent of the observed structure factors were not included in the refinement and were instead used for validation by free R-factor calculations. The details of the refinement statistics are shown in Table 2. Molecular graphics were produced using ChimeraX [46].

## Results

### Construction and validation of a high-throughput screening system

**Fig. 2:**
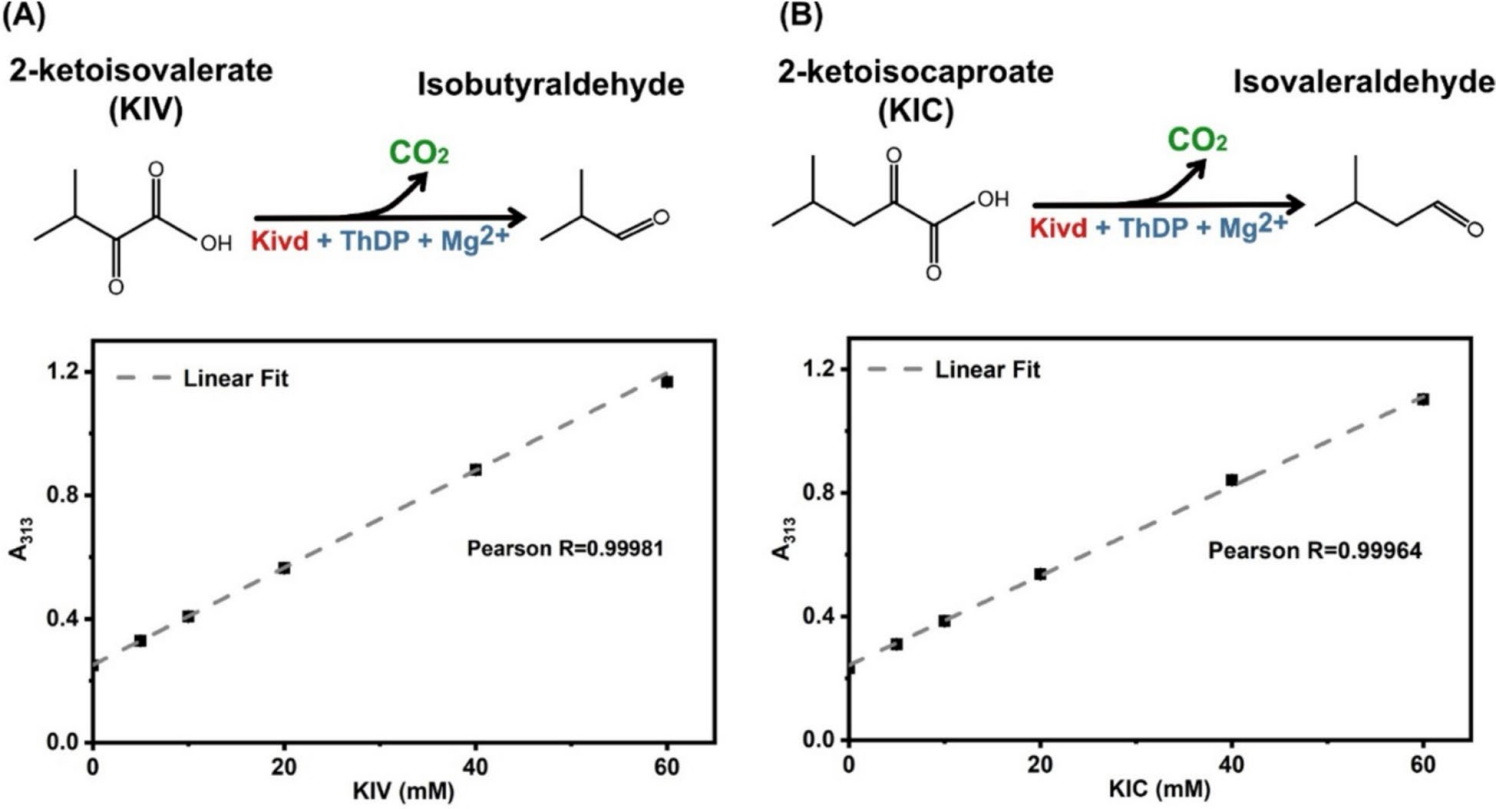
Initial conception of a high-throughput screening method based on substrate consumption assessed via absorbance measurement at 313 nm (A_313_). **a** Conversion of 2-ketoisovalerate (KIV) to isobutyraldehyde by Kivd, with ThDP and Mg^2+^ as cofactors. Correlation curve between KIV concentrations and A_313_ readings. **b** Conversion of 2-ketoisocaproate (KIC) to isovaleraldehyde by Kivd, with ThDP and Mg^2+^ as cofactors. Correlation curve between KIC concentrations and A_313_ readings. Results represent the mean of three technical replicates. Error bars represent standard deviation

Kivd^S286T^ is a key enzyme in the 2-keto acid pathway, catalyzing the conversion of 2-ketoisovalerate (KIV) and 2-ketoisocaproate (KIC) to isobutyaldehyde and isovaleraldehyde, respectively, in *Synechocystis* sp. PCC 6803 (*Synechocystis*). Kivd^S286T^ variants exhibiting enhanced performance may lead to increased substrate consumption and product formation rates. A spectral scan revealed that both KIV and KIC have an absorbance peak at 313 nm, indicating that a decrease in absorbance at this wavelength could serve as an indicator of substrate consumption. As illustrated in Fig. 2, there is a strong correlation between absorbance at 313 nm (A_313_) and KIV or KIC concentrations. The A_313_ increased linearly with KIV or KIC concentrations ranging from 0 to 60 mM.

**Fig. 3:**
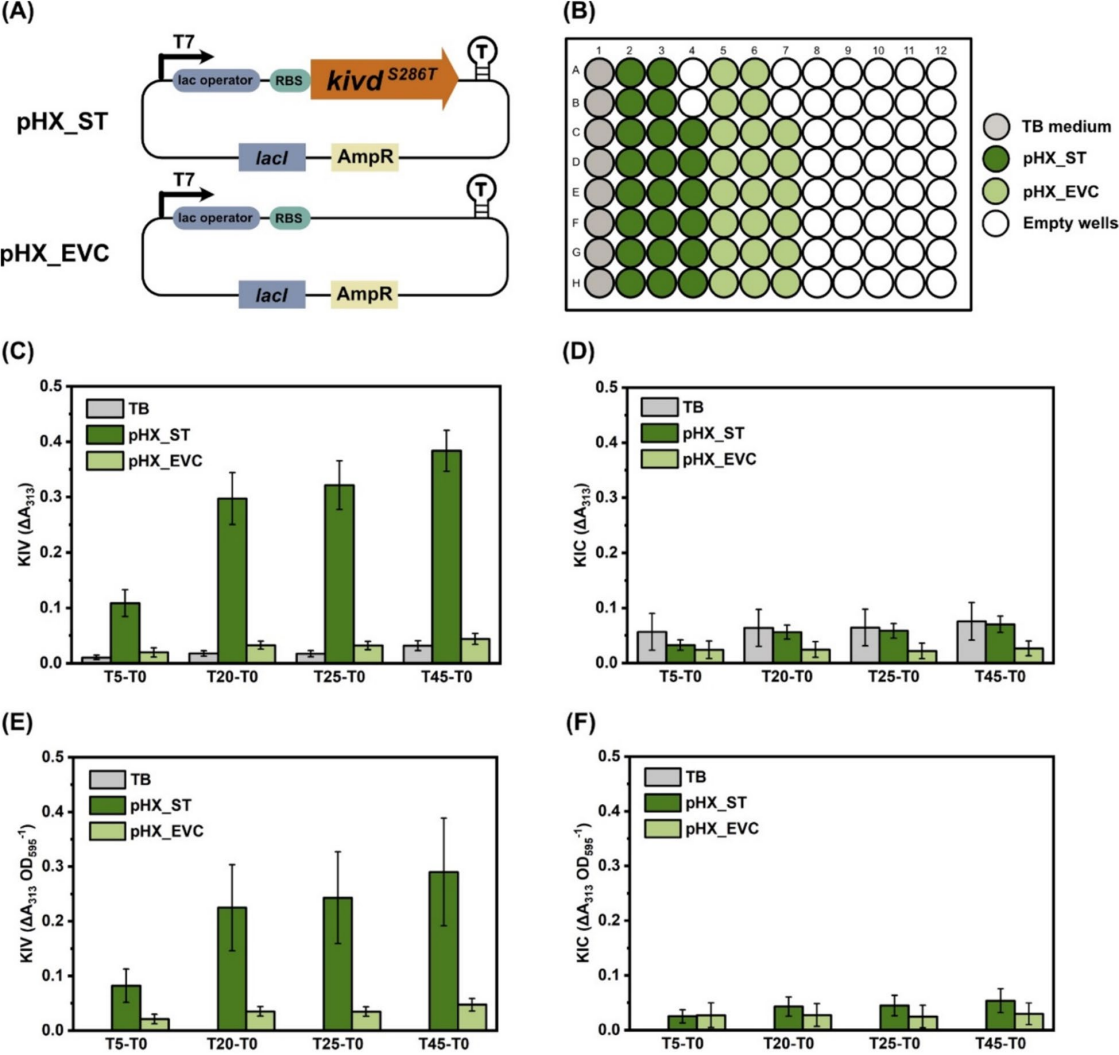
Development and validation of the high-throughput screening method using the supernatant of crude extracts from two *E. coli* strains HXEC_ST and HXEC_EVC. The change of A_313_ indicates the consumption of 2-ketoisovalerate (KIV) or 2-ketoisocaproate (KIC). **a** Schematic presentation of the genetic constructs used to generate the engineered *E. coli* strains HXEC_ST and HXEC_EVC. **b** Layout of 96-well plate for the enzymatic assay supplemented with crude protein extract supernatant from strain HXEC_ST or HXEC_EVC. Wells with TB medium addition serve as controls. **c** Absorbance changes at 313 nm after incubating enzymatic reactions for 5 h, 20 h, 25 h, or 45 h. 60 mM KIV were added in the enzymatic reaction. **d** Absorbance changes at 313 nm after incubating enzymatic reactions for 5 h, 20 h, 25 h, or 45 h. 60 mM KIC were added in the enzymatic reaction. **e** Absorbance changes at 313 nm per OD_595_ after incubating enzymatic reactions for 5 h, 20 h, 25 h, or 45 h. 60 mM KIV were added in the enzymatic reaction. **f** Absorbance changes at 313 nm per OD_595_ after incubating enzymatic reactions for 5 h, 20 h, 25 h, or 45 h. 60 mM KIC was added in the enzymatic reaction. Results represent the mean of 8 or 22 biological replicates. Error bars represent standard deviation

Next, it is crucial to test and validate the proposed activity assay based on A_313_ measurement for direct integration into a high-throughput screening system to identify Kivd^S286T^ variants with improved catalytic activity. For this purpose, two *E. coli* strains, HXEC_ST and HXEC_EVC, were constructed by transforming plasmids pHX_ST and pHX_EVC into *E. coli* BL21 (DE3), respectively (Fig. 3a). Successful expression of Kivd^S286T^ protein in the HXEC_ST strain was confirmed (Fig. S2). Single colonies of both strains were inoculated in a 96-DW plate according to the layout shown in Fig. 3b. A_313_ was measured before the reaction and at 5, 20, 25, and 45 h after the reaction started. As anticipated, the control group, which did not contain any crude protein, exhibited negligible changes in A_313_ (Fig. 3c, d). When KIV was used as the substrate, reactions with crude Kivd^S286T^ protein showed a decrease in A_313_ of 0.11 after 5 h of incubation, which further decreased to 0.30 after 20 h of incubation (Fig. 3c). Conversely, reactions with crude protein from the HXEC_EVC strain showed only a slight decrease in A_313_, with a reduction of only 0.03 after 20 h of incubation. Similar patterns were observed after normalization on a per OD_595_ basis (Fig. 3e). However, when KIC was used as substrate, no clear differences in A_313_ changes were observed across different groups, even after 45 h of incubation (Fig. 3d, f).

To conclude, the A_313_-based activity assay proved effective for detecting KIV substrate consumption but not for KIC substrate (Fig. 3). Therefore, subsequent high-throughput screening of Kivd^S286T^ variants with better performance will focus exclusively on KIV substrate consumption. The KIV consumption will be assessed using the ΔA_313_ (start-point A_313_ − end-point A_313_), as consistent results were observed before and after OD_595_ normalization (Fig. 3c, f).

### Superior Kivd^S286T^ variants identification via a high-throughput screening system

**Fig. 4:**
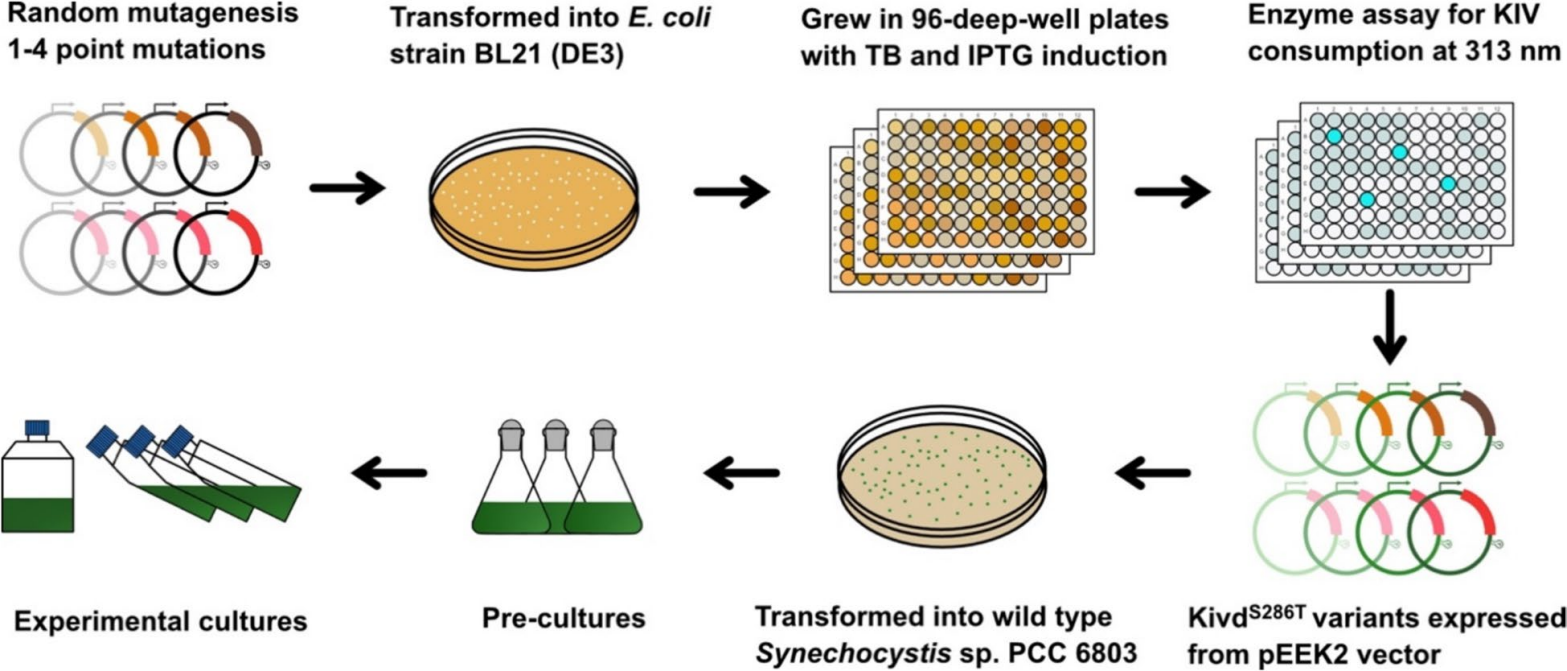
Directed evolution of Kivd^S286T^ using high-throughput screen method. The mutant library was constructed via error-prone PCR to introduce 1–4 point substitutions on the *kivd*^*S286T*^ gene fragment. Subsequently, the mutant library was transformed into *E. coli* strain BL21 (DE3), and single colonies were cultivated in 96-deep-well plates. Crude cell supernatant was utilized in the developed activity assay for screening purpose. The Kivd^S286T^ variants exhibited improved performance in the assay were cloned into the pEEK2 vector and transformed into wild-type *Synechocystis* sp. PCC 6803 for IB and 3M1B production characterization

The high-throughput screening pipeline is outlined in Fig. 4. Four independent random mutagenesis libraries of Kivd^S286T^ were generated by error-prone PCR, introducing 1–4 point substitutions per gene. The resulting mutagenesis libraries were cloned into an expression vector, which was then used to transform *E. coli* BL21 (DE3). For each library, 8–12 transformants were randomly selected and sequenced for library quality assessment. The proportion of Kivd^S286T^ variants containing 1–4 point substitutions across the four libraries ranged from 37.5 to 100% (Fig. S3). In total, 1600 Kivd^S286T^ variants, with 400 variants from each library, were selected and screened using the developed high-throughput system.

**Fig. 5:**
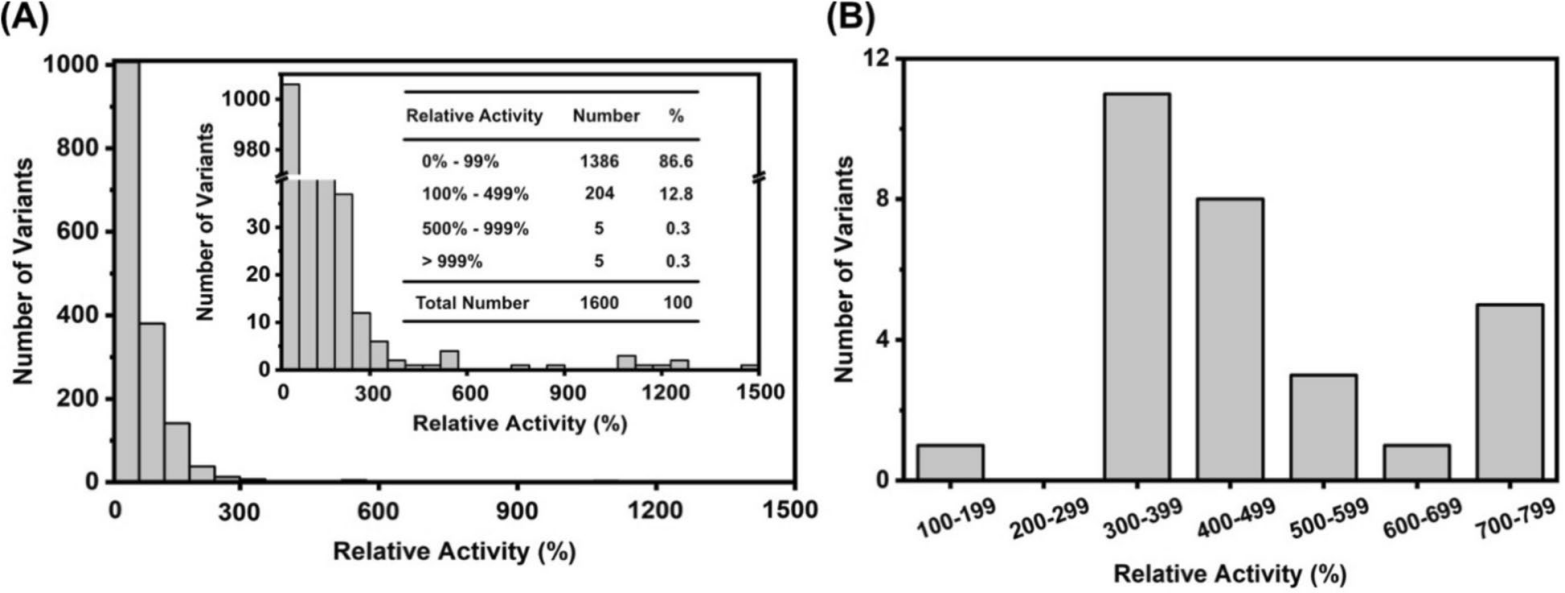
Identification of Kivd^S286T^ variants with improved performance using the high-throughput screen method. **a** Distribution of relative activity of Kivd^S286T^ variants compared to the original Kivd^S286T^ after incubating enzymatic reactions for 2 h. 60 mM KIV were added prior to starting enzymatic reaction. **b** Re-examination of the 29 best-performing Kivd^S286T^ variants from the library screen, with three technical replicates. Distribution of relative activity compared to Kivd^S286T^ after incubating enzymatic reactions for 2 h. 60 mM KIV was added prior to starting each enzymatic reaction

The relative activity of the screened Kivd^S286T^ variants, represented as a percentage of the original Kivd^S286T^ activity, displayed a broad distribution, ranging from 0 to 1500% (Fig. 5a). Of the 1600 variants tested, 214 variants showed improved activity, including 10 variants that exhibited a more than fivefold increase in activity. Notably, the majority of the Kivd^S286T^ variants exhibited reduced activity or complete loss of catalytic function compared to the original Kivd^S286T^. To minimize false positives in subsequent analyses, the 29 Kivd^S286T^ variants with the highest relative activity were retested in technical triplicates. All of these variants demonstrated significantly higher activity than the original Kivd^S286T^, with over 96% exhibiting relative activity exceeding 300% (Fig. 5b). The consistency of results across the two rounds of activity testing underscores the efficacy and reproducibility of the developed high-throughput screening system.

### Isobutanol (IB) and 3-methyl-1-butanol (3M1B) production in *Synechocystis* using identified Kivd^S286T^ variants

**Fig. 6:**
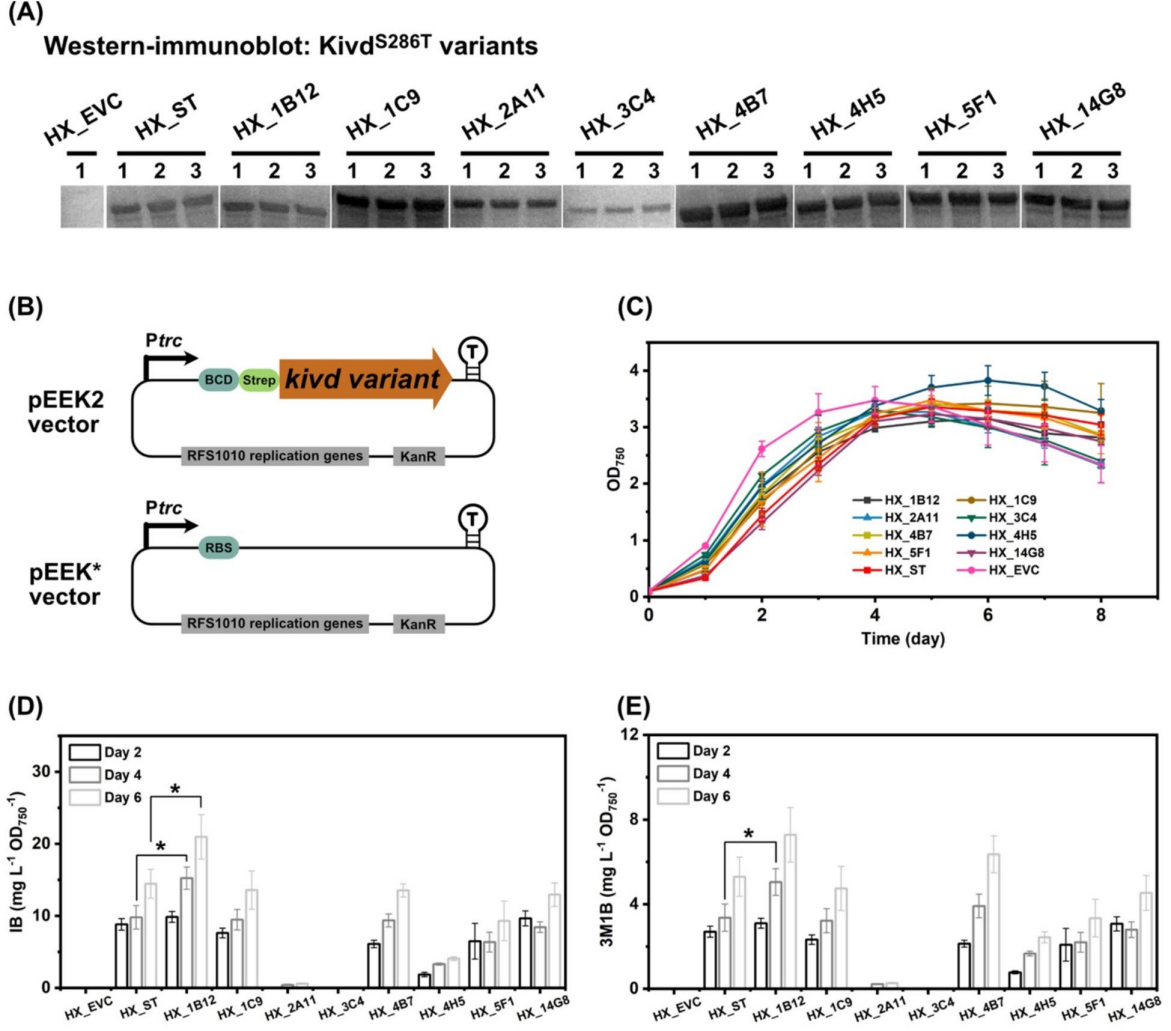
Engineered *Synechocystis* sp. PCC 6803 strains with heterologous expression of identified Kivd^S286T^ variants from high-throughput screening. **a** Western-immunoblot detection of Kivd^S286T^ variants. Five micrograms of total soluble proteins were loaded for each strain to detect Strep-tagged Kivd^S286T^ variants. Protein size: 61 kDa. **b** Schematic presentation of two vectors, pEEK2 and pEEK*. pEEK2 vector was used to express the identified Kivd^S286T^ variants in *Synechocystis* cells. pEEK* vector was used to generate a control strain, designated as HX_EVC, without Kivd^S286T^ protein expressed. See Table 1 for details of the strains. BCD, bicistronic design. **c** Growth profile of engineered *Synechocystis* strains. **d** Isobutanol (IB) production per OD_750_ of engineered strains on days 2, 4, and 6. **e** 3-methyl-1-butanol (3M1B) production per OD_750_ of engineered strains on days 2, 4, and 6. Results represent the mean of three biological replicates. Error bars represent standard deviation. The asterisk represents significant difference between strains (*t* test, *p < 0.05)

From the 1600 Kivd^S286T^ variants screened, the eight best-performing variants were selected for sequencing. The point substitutions in these selected Kivd^S286T^ variants are detailed in Table 3, showing distribution across the entire protein sequence. These eight variants, along with the original Kivd^S286T^, were successfully expressed in a wild-type *Synechocystis* strain using self-replicating plasmid pEEK2 (Fig. 6a), resulting in the generation of nine engineered *Synechocystis* strains (Table 1). Expression of Kivd variants was driven by the P*trc* promoter, coupled with a bicistronic design (BCD) element [47]. In addition, a control strain carrying pEEK* plasmid was generated (Fig. 6b, Table 1).

**Table 3:** Kivd^S286T^ variants with mutations

Kivd^S286T^ variants	Mutation sites	Relative activity (%)
ST	S286T	100 ± 0
3C4	S286T, A116G, V160L, K299R, L441P	464 ± 129
1C9	S286T, L261L (CTG–CTA)	471 ± 35
14G8	S286T, L77L (CTG–TTG), A152V, K497E	537 ± 178
1B12	S286T, T186S, K419E	658 ± 296
2A11	S286T, L353M, T407S, E438G, R444C, S486S (AGC–AGT)	746 ± 147
4B7	S286T, Y406F	767 ± 301
4H5	S286T, K158R, L221M, T372T (ACC–ACA), F388L, K392N, T407S, K510N, Y520H	785 ± 176
5F1	S286T, A116G, K121N, N265D, I276T	844 ± 363
T186S	S286T, T186S	/
K419E	S286T, K419E	/

Distinct growth patterns were observed among the strains expressing different Kivd^S286T^ variants (Fig. 6c). Notably, the control strain HX_EVC, which did not express any Kivd^S286T^ variants, exhibited the highest OD_750_ value between days 0 and 4 (Fig. 6c). Among the eight Kivd^S286T^ variants tested, only the strain HX_1B12 demonstrated a significant improvement in IB and 3M1B production per OD_750_ (Fig. 6d, e). On day 4, strain HX_1B12 exhibited 55% and 50% increase in IB and 3M1B production, respectively, compared to the control strain HX_ST. In contrast, strains HX_2A11, HX_3C4, and HX_4H5 exhibited significantly lower IB and 3M1B production per OD_750_ relative to strain HX_ST, while the remaining strains produced IB and 3M1B at levels comparable to strain HX_ST (Fig. 6d, e).

### Further characterization of 1B12 variant

One Kivd^S286T^ variant, 1B12, demonstrated a significant improvement in IB and 3M1B production per OD_750_ in *Synechocystis* (Fig. 6). This variant contained two point substitutions, T186S and K419E. To assess individual contribution of these point substitutions to the observed production enhancement, two new Kivd^S286T^ variants named T186S and K419E, each carrying one of the point substitutions, were generated. Subsequently, two new engineered *Synechocystis* strains, HX_T186S and HX_K419E, were constructed (Table 1).

**Fig. 7:**
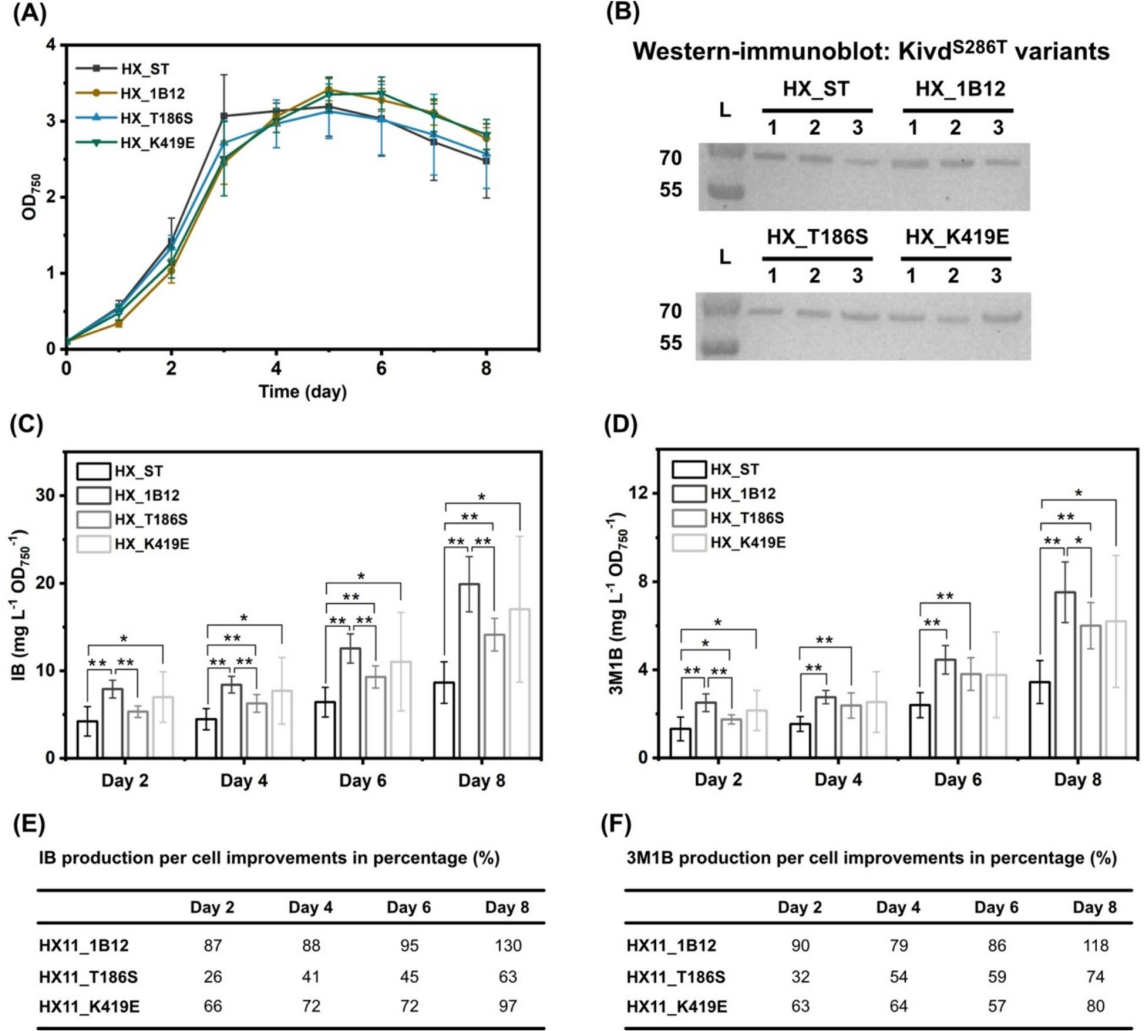
Further investigation of Kivd^S286T^ variant 1B12 by generating two novel variants containing single point substitution derived from 1B12, designated as T186S and K419E. **a** Growth profile of engineered *Synechocystis* sp. PCC 6803 strains HX_ST, HX_1B12 (T186S, K419E), HX_T186S, and HX_K419E. **b** Western-immunoblot detection of Kivd^S286T^ variants. Five micrograms of total soluble proteins were loaded for each strain to detect Strep-tagged Kivd^S286T^ variants. Protein size: 61 kDa. **c** Isobutanol (IB) production per OD_750_ of engineered strains on days 2, 4, 6, and 8. **d** 3-methyl-1-butanol (3M1B) production per OD_750_ of engineered strains on days 2, 4, 6, and 8. **e** IB production per OD_750_ improvements in percentage (%). **f** 3M1B production per OD_750_ improvements in percentage (%). Results represent the mean of three biological replicates with three technical replicates. Error bars represent standard deviation. The asterisk represents significant difference between strains (*t* test, *p < 0.05, **p < 0.005)

During 8-day cultivation, no significant growth differences were observed among the four strains (HX_ST, HX_1B12, HX_T186S, and HX_K419E) tested, likely due to variations among the biological triplicates of each strain (Fig. 7a). Protein expression of Kivd^S286T^ variants was successfully detected and exhibited similar patterns across the strains (Fig. 7b). Interestingly, both strains HX_ST and HX_1B12 exhibited batch-dependent variations in IB and 3M1B production levels (Figs. 6 and 7). These differences may stem from different factors: variations in cell growth conditions between experimental batches, temporal difference in product accumulation, or experimental variability. Compared to the control strain HX_ST, strain HX_T186S showed significantly higher IB production per OD_750_ on days 4, 6, and 8, as well as significantly higher 3M1B production per OD_750_ on days 2, 4, 6, and 8, (Fig. 7c, d). The maximal enhancement observed in strain HX_T186S were 63% for IB and 74% for 3M1B on day 8 (Fig. 7e, f). Strain HX_K419E also exhibited significantly higher IB production per OD_750_ on days 2, 4, 6, and 8, while significantly higher 3M1B production per OD_750_ was observed on days 2 and 8 (Fig. 7c, d). The maximal enhancements achieved by strain HX_K419E were 97% for IB and 80% for 3M1B on day 8 (Fig. 7e, f). Compared to strain HX_1B12, HX_T186S produced significantly less IB production per OD_750_ on days 2, 4, 6, and 8, and significantly less 3M1B production per OD_750_ on days 2 and 8 (Fig. 7c, d). For strain HX_K419E, there was no significant difference in IB and 3M1B production per OD_750_ compared to strain HX_1B12, likely due to the relatively large variations of production levels in strain HX_K419E. In conclusion, the point substitutions, T186S and K419E, exhibit synergetic effects enhancing both IB and 3M1B production per OD_750_ in *Synechocystis*.

### Structure of Kivd^S286T^

**Fig. 8:**
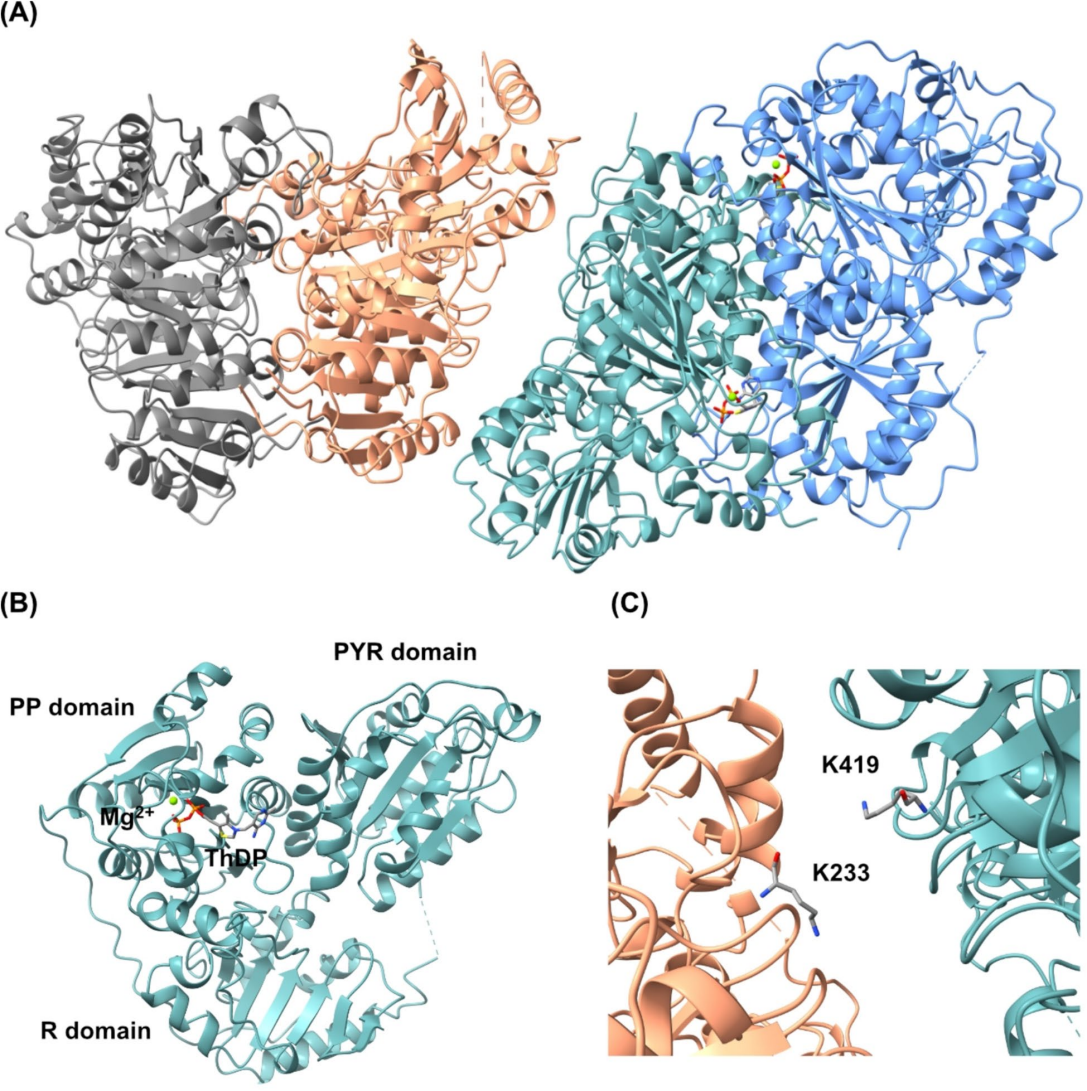
Overall structure of Kivd^S286T^. **a** Crystal structure of Kivd^S286T^ is shown as a ribbon model. The tetramer structure consists of four chains. **b** Monomer structure of Kivd^S286T^, consisting of PP domain, PYR domain, and R domain. ThDP is shown as stick model and Mg^2+^ is colored light green. **c** Closeup view of the Kivd^S286T^ dimer–dimer interface. Residues K419 and K233 are shown as sticks. Chain A, blue–green; Chain B, light blue; Chain C, orange; Chain D, gray

The 2.8 Å X-ray crystal structure of Kivd^S286T^ reveals a tetrameric assembly composed of four polypeptide chains (Fig. 8a). The tetrameric structure is consistent with the results obtained from gel filtration chromatography, confirming that the crystal structure of Kivd^S286T^ represents the biologically active form of the protein. Due to low electron density in the X-ray crystallographic data, several disordered regions were not modeled. These regions include residues 181–187 in chains A and B, and residues 180–195 in chains C and D. In addition, residues from 456 to 477 and from 529 to 548 in the C-terminal region of chains C and D were not included in the model. Notably, the ThDP and Mg^2^⁺ cofactors are clearly resolved within the dimer formed by chains A and B, while these cofactors are absent in the dimer of chains C and D (Fig. 8a).

Each Kivd^S286T^ monomer consists of three distinct domains (Fig. 8b). The N-terminal PYR domain binds the pyrimidine ring of ThDP, the central R domain lacks bound cofactors, and the C-terminal PP domain interacts with the diphosphate group of ThDP. The enzyme’s active site is located at the interface between the PP and PYR domains of adjacent monomers. The ThDP cofactor, crucial for the decarboxylation reaction, is anchored within the active site through interactions with the conserved GDGX25-30NN motif [48], which coordinates with the diphosphate tail and an Mg^2+^ ion (Fig. 8a, b).

## Discussion

The high-throughput screening system developed in this study utilizes absorbance change at 313 nm as an indicator of Kivd activity, effectively monitors KIV consumption but not KIC. This outcome is consistent with the substrate preference of Kivd^S286T^ enzyme, which favors KIV over KIC for enzymatic reactions [14, 18]. Consequently, the screening assay lacks sensitivity in detecting KIC consumption, at least within the tested incubation period of 45 h. The slight decrease in A_313_ observed in reaction samples lacking Kivd^S286T^ may be attributed to the spontaneous degradation of KIV and KIC during incubation at 30 °C. However, this minor degradation has negligible impacts on its application in high-throughput screening of Kivd^S286T^ variants with enhanced catalytic activity.

To maximize the proportion of active enzymes in the random mutagenesis libraries, point substitutions in the original Kivd^S286T^ were limited to 1–4. However, the proportion of Kivd^S286T^ variants containing 1–4 point substitutions varied between 37 and 100% across four independent mutagenesis libraries. This inter-library variability may arise from limited sequencing depth, as small sample size can lead to stochastic sampling bias. In addition, differences in mutation efficiency across libraries may contribute to the observed inconsistencies in mutation distribution. Among Kivd^S286T^ variants generated by error-prone PCR, 86.6% exhibited reduced activity or complete loss of activity compared to the original Kivd^S286T^ (Fig. 5). These findings are consistent with previous studies on protein directed evolution, which suggest that point substitutions caused by random mutation are often deleterious, resulting in the majority of protein variants showing reduced activity, with only 0.01–1% being beneficial [24, 49, 50].

After testing the top eight promising variants from random mutagenesis library, one variant, 1B12, exhibited significantly improved IB and 3M1B production per OD_750_ in *Synechocystis* sp. PCC 6803 (*Synechocystis*) compared to the original Kivd^S286T^ (Fig. 6). Similar protein expression levels were detected for original Kivd^S286T^ and 1B12 in *Synechocystis* (Fig. 6a), indicating that the observed increase in IB and 3M1B production per OD_750_ was due to improved catalytic activity rather than elevated protein expression. Variant 1B12 contains two point substitutions, K419E and T186S, both of which contribute to the enhanced IB and 3M1B production. Based on the tetrameric crystal structure of Kivd^S286T^ (Fig. 8a), one dimer contains the essential cofactors ThDP and Mg^2+^, which are required for the catalytic function of Kivd^S286T^. Interestingly, the adjacent dimer lacks these cofactors. The substitution of lysine at residue K419 with glutamate, located at the dimer–dimer interface (Fig. 8c), represents a critical alteration. This charge reversal likely affects the conformation of the dimer–dimer interface, potentially modulating enzymatic activity. On the other hand, the introduction of a negatively charged glutamate at position 419 may facilitate the formation of a stabilizing salt bridge with residue K233 from the adjacent dimer, thereby impacting dimer–dimer interactions and, consequently, enzyme functionality. Further investigations are required to fully elucidate the impact of this residue substitution on catalytic performance. In contrast, residue T186 is located within a flexible loop, whose precise positioning within the tetrameric structure remains challenging to determine. Flexible loops play a crucial role in protein function through various mechanisms [51–53]. Although distant from the active sites, this flexible loop connects the catalytic PYR domain and the regulatory R domain, which may function as an allosteric switch. As one hypothesis, the T186S substitution may disrupt the interaction between the flexible loop and an inhibitory factor, thereby enhancing catalytic activity through allosteric regulation.

Among the tested Kivd^S286T^ variants that did not show enhanced production in *Synechocystis*, variations in protein expression were observed (Fig. 6). Notably, variant 1C9, which has a synonymous mutation at L261 (CTG to CTA), exhibited significantly higher protein expression. Despite this increase, IB and 3M1B production per OD_750_ remained comparable to that of the original Kivd^S286T^ (Fig. 6a, d, e). This discrepancy might be attributed to the codon usage preference of *Synechocystis* [54]. However, the exact mechanism underlying the inconsistency between protein expression and IB/3M1B production in strain HX_1C9 remains unclear. In addition, four other Kivd^S286T^ variants (4B7, 4H5, 5F1, and 14G8) exhibited higher protein expression levels but did not produce increased IB and 3M1B production per OD_750_ compared to the original Kivd^S286T^ (Fig. 6). These findings suggest that the point substitutions in these variants may have increased protein stability at the expense of catalytic activity, consistent with previous reports of the trade-off between protein stability and protein activity [55, 56]. Enhancing enzyme stability or expression while maintaining or improving its catalytic activity is inherently challenging [57, 58]. Variant 3C4 showed a marked decrease in protein expression (Fig. 6a) and failed to produce any IB or 3M1B (Fig. 6d, e), indicating that the point substitutions in variant 3C4 affected both protein expression level and catalytic function. Interestingly, similar protein expression for Kivd^S286T^ variants was observed for strain HX_2A11 and HX_ST, though IB and 3M1B was barely produced by HX_2A11 (Fig. 6).

The proposed high-throughput screening system successfully identifies Kivd^S286T^ variants with better performance for IB and 3M1B production in *Synechocystis*, albeit with relatively low efficiency. The current screening method relies on substrate consumption, which poses a certain limitation: the selected variants may be active in consuming KIV but may not necessarily produce IB; they can instead produce other chemicals. To address this limitation, developing a screening method based on product formation would be a more targeted approach. The established high-throughput screening uses crude protein extracts from *E. coli* for activity assay, and the identified Kivd^S286T^ variants were subsequently introduced into *Synechocystis* for production testing. The metabolic differences between *E. coli* and *Synechocystis* may lead to varying performance of Kivd^S286T^ variants in these two organisms.

To assess whether in vitro enzyme activity correlates with in vivo production in *E. coli*, we tested the selected eight Kivd^S286T^ variants in *E. coli* for IB and 3M1B production. However, none of the selected variants improved IB or 3M1B production per OD_595_ (Fig. S4), indicating a poor correlation. One possible explanation is that the tightly controlled reaction conditions of in vitro high-throughput screening may not reflect the complex intracellular environment, leading to discrepancies in the performance of mutated variants. For further optimization, it would be beneficial to apply high-throughput screening directly in *Synechocystis*. However, implementing high-throughput screening in *Synechocystis* with the current method present challenges. As an alternative, biosensor-assisted screening, which has been successfully applied in various organisms to optimize bioproduction [59–62], could be explored.

## Conclusions

In summary, this is the first demonstration of performing directed evolution on a bottleneck enzyme to increase chemical production in cyanobacteria. A high-throughput screening method was successfully established by measuring absorbance change at 313 nm to monitor the consumption of the substrate 2-ketoisovalerate. Screening 1600 variants from four independent libraries led to the identification of a Kivd^S286T^ variant (1B12) featuring dual T186S and K419E substitutions. This variant exhibited a significant increase in IB and 3M1B production per OD_750_. In addition, the X-ray crystal structure if Kivd^S286T^ was determined at a resolution of 2.8 Å. This crystal structure not only provides insights into the improved performance of the identified Kivd^S286T^ variant, but also offers a foundation for further rational design of Kivd^S286T^ to improve diverse properties, for example, protein stability, activity, and substrate selectivity.

## Supplementary Information

Additional file 1.

## Data Availability

Full wwPDB X-ray Structure Validation Report (PDB ID: 9MSA) Alpha-ketoisovalerate decarboxylase (Kivd) from Synechocystis sp. PCC 6803 with substitution S286T attached as “D-1000291567-ValidationReport-KivdS286T.pdf”.

## Abbreviations

3M1B: 3-Methyl-1-butanol
A_313_: Absorbance at 313 nm
Adh: Alcohol dehydrogenase
AlsS: Acetolactate synthase
AmpR: Ampicillin resistance cassette
DCM: Dichloromethane
*E.** coli*: *Escherichia coli*
EP-PCR: Error-prone PCR
IB: Isobutanol
IlvC: Acetohydroxy-acid isomeroreductase
IlvD: Dihydroxy-acid dehydratase
IMAC: Immobilized metal affinity chromatography
IPTG: Isopropyl-β-D-thiogalactopyranoside
KIC: 2-Ketoisocaproate
KIV: 2-Ketoisovalerate
Kivd: *α*-Ketoisovalerate decarboxylase
KmR: Kanamycin resistance cassette
LB: Lysogeny broth
LeuA: 2-Isopropylmalate synthase
LeuB: 3-Isopropylmalate dehydrogenase
LeuCD: 3-Isopropylmalate dehydratase
*Synechocystis*: *Synechocystis* Sp. PCC 6803
T: Terminator BBa_B0015
TB: Terrific broth
ThDP: Thiamine diphosphate
