# Supplementary material for: Directed evolution of α-ketoisovalerate decarboxylase for improved isobutanol and 3-methyl-1-butanol production in cyanobacteria
Source: Biotechnol Biofuels Bioprod. 2025 Jul 31;18:84. doi: 10.1186/s13068-025-02687-6 (PMC12312269; doi:10.1186/s13068-025-02687-6)
Supplement: Supplementary file 1 — Additional file 1. [file 13068_2025_2687_MOESM1_ESM.docx]

**Directed evolution of α-ketoisovalerate decarboxylase for improved isobutanol and 3-methyl-1-butanol production in cyanobacteria**

Hao Xie^1,2^, Afshan Begum^3^, Laura H. Gunn^3,4^ and Peter Lindblad^1^*

^1^Microbial Chemistry, Department of Chemistry-Ångström Laboratory, Uppsala University, Uppsala, Sweden

^2^College of Bioengineering, Sichuan University of Science & Engineering, Yibin, Sichuan, China

^3^Department of Cell and Molecular Biology, Uppsala University, Uppsala, Sweden

^4^Plant Biology Section, Cornell University, Ithaca, New York, USA

*Corresponding author: Peter Lindblad, peter.lindblad@kemi.uu.se

**Additional file 1**

**Figures S1 - S4**

**Tables S1 - S2**


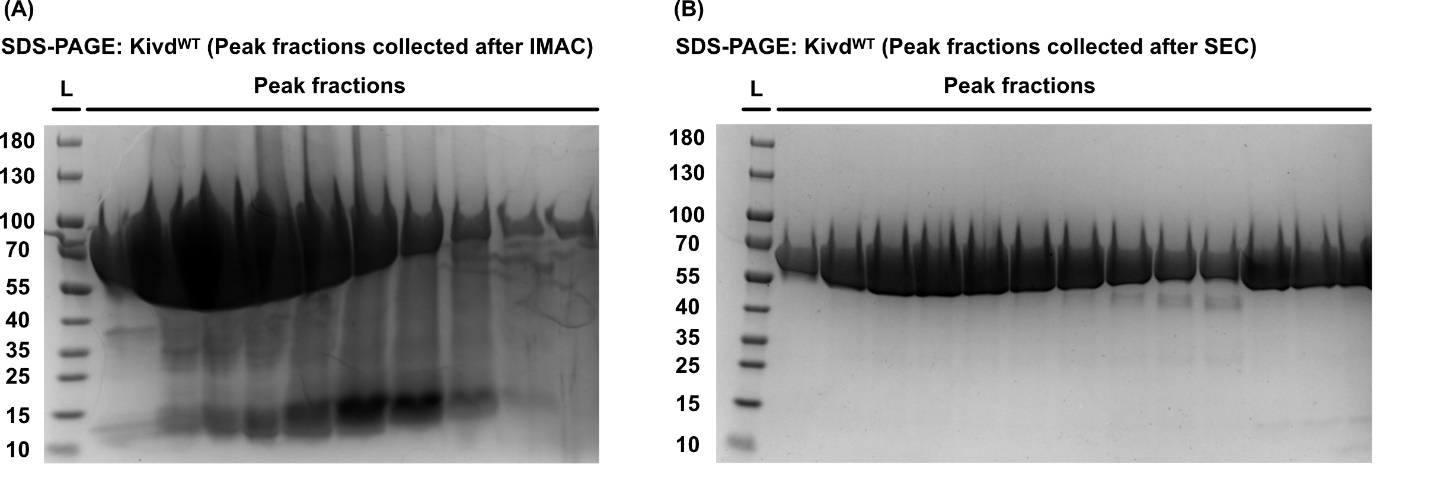


**Fig. S1**: SDS-PAGE analysis of protein Kivd^WT^ in different steps during purification. **(A)** SDS-PAGE analysis of Kivd^WT^ with a H_6_-Ub tag after IMAC (immobilized metal affinity chromatography) step **(B)** SDS-PAGE analysis of Kivd^WT^ without a H_6_-Ub tag after SEC (size-exclusion chromatography) step. Five microliter from each fraction were loaded for analysis. L, ladder (in kDa). Protein size: Kivd^WT^ with H_6_-Ub tag, 72 kDa; Kivd^WT^ without H_6_-Ub tag, 61 kDa.


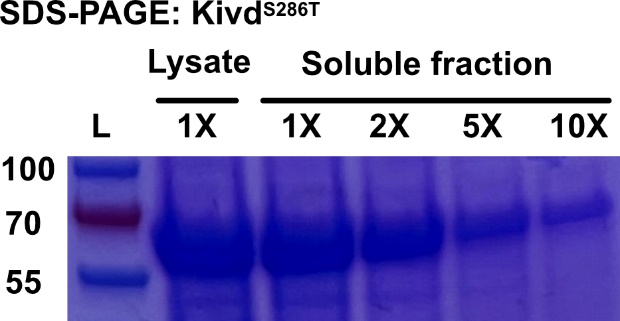


**Fig. S2**: SDS-PAGE analysis of the recombinant protein Kivd^S286T^ extracted from *E. coli* BL21(DE3) cells. Cell pellet from 1 mL of cell culture was lysed with 100 μL B-PER complete reagent. The resulting lysate and the soluble fraction after centrifugation were loaded for analysis. For soluble fraction, four different dilutions were applied. 1X, no dilution; 2X, two-time dilution; 5X, five-time dilution; 10X, ten-time dilution. L, ladder (in kDa). Protein size: Kivd^S286T^, 61 kDa.


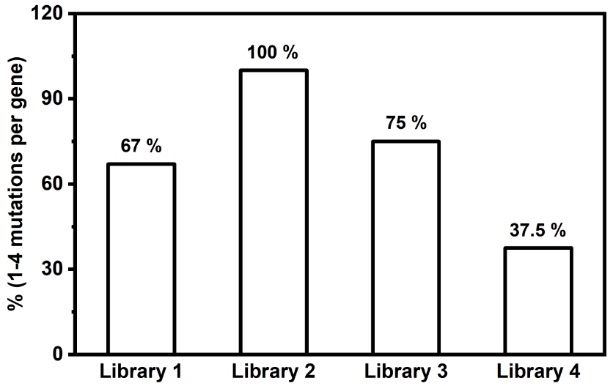


**Fig. S3**: Four individual random mutagenesis libraries constructed for high-throughput screening. The percentage of *kivd^S286T^* variants with 1 - 4 point substitutions are presented for individual libraries.


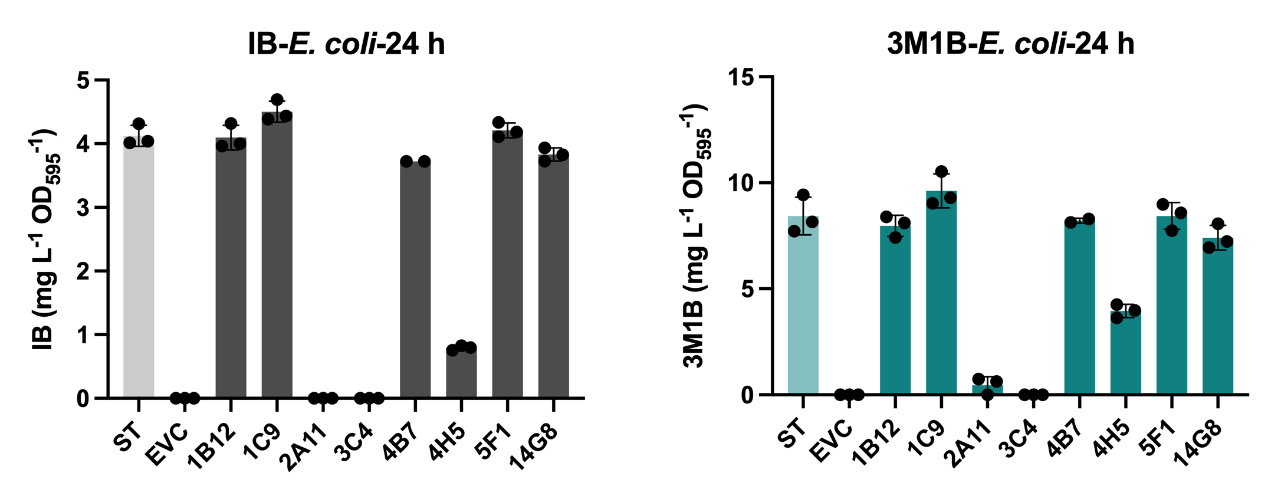


**Fig. S4**: Isobutanol (IB) and 3-methyl-1-butanol (3M1B) production per OD_595_ of engineered *E. coli* strains after 24 h cultivation. Results represent the mean of three biological replicates. Error bars represent standard deviation.

**Table S1.** Plasmid used in this study. Expressed genes in bold.

| **Plasmid** | **Relevant characteristics^a^** | **Reference** |
| --- | --- | --- |
| pHUE | pHUE_T7_RBS_H6-Ub_T_AmpR | [1] |
| pUC57_kivd | pUC57_RBS_H6-Ub_***kivd***_AmpR | This study |
| pUC57_ST | pUC57_RBS_H6-Ub_***kivd^S286T^***_AmpR | This study |
| pHX_EVC | pHX_T7_RBS_T_AmpR | This study |
| pHX_ST | pHX_T7_RBS_ ***kivd^S286T^***_T_AmpR | This study |
| pHX_1B12 | pHX_T7_RBS_***1B12***_T_AmpR | This study |
| pHX_1C9 | pHX_T7_RBS_***1C9***_T_AmpR | This study |
| pHX_2A11 | pHX_T7_RBS_***2A11***_T_AmpR | This study |
| pHX_3C4 | pHX_T7_RBS_***3C4***_T_AmpR | This study |
| pHX_4B7 | pHX_T7_RBS_***4B7***_T_AmpR | This study |
| pHX_4H5 | pHX_T7_RBS_***4H5***_T_AmpR | This study |
| pHX_5F1 | pHX_T7_RBS_***5F1***_T_AmpR | This study |
| pHX_14G8 | pHX_T7_RBS_***14G8***_T_AmpR | This study |
| pEEK* | pEEK_P*trc*_RBS_T_KanR | Englund, E., unpublished |
| pEEK2_ST | pEEK2_P*trc*BCD_***Strep-ST***_T_KanR | This study |
| pEEK2_1B12 | pEEK2_P*trc*BCD_***Strep-1B12***_T_KanR | This study |
| pEEK2_1C9 | pEEK2_P*trc*BCD_***Strep-1C9***_T_KanR | This study |
| pEEK2_2A11 | pEEK2_P*trc*BCD_***Strep-2A11***_T_KanR | This study |
| pEEK2_3C4 | pEEK2_P*trc*BCD_***Strep-3C4***_T_KanR | This study |
| pEEK2_4B7 | pEEK2_P*trc*BCD_***Strep-4B7***_T_KanR | This study |
| pEEK2_4H5 | pEEK2_P*trc*BCD_***Strep-4H5***_T_KanR | This study |
| pEEK2_5F1 | pEEK2_P*trc*BCD_***Strep-5F1***_T_KanR | This study |
| pEEK2_14G8 | pEEK2_P*trc*BCD_***Strep-14G8***_T_KanR | This study |
| pEEK2_T186S | pEEK2_P*trc*BCD_***Strep-T186S***_T_KanR | This study |
| pEEK2_K419E | pEEK2_P*trc*BCD_***Strep-K419E***_T_KanR | This study |
| pHUE_kivd | pHUE_T7_RBS_H6-Ub_***kivd***_T_AmpR | This study |
| PHUE_ST | pHUE_T7_RBS_H6-Ub_***kivd^S286T^***_T_AmpR | This study |

a KmR, kanamycin resistance cassette; AmpR, ampicillin resistance cassette; T, Terminator BBa_B0015

**Table S2.** Oligonucleotides used in this study.

| **Primer name** | **Oligonucleotide sequence** |
| --- | --- |
| **A. Primers for fragment amplification** | |
| pHX_F | Pho-CTAGCATAACCCCTTGGGG |
| pHX_R | Pho-TCTCCTTCTTAAAGTTAAACAAAATT |
| pHUE_F | CGGAGCAAAATAAAAGCTAAGAATTCGAGCTCGGTACCGTC |
| pHUE_R | GTAGTCGCCAACGGTGTACATGGTATATCTCCTTCTTAAAG |
| kivd_F | ATGTACACCGTTGGCGACTAC |
| kivd_R | TTAGCTTTTATTTTGCTCCG |
| kivd_BglII_F | TATAAGATCTATGTACACCGTTGGCGACTAC |
| kivd_SpeI_R | TATAACTAGTTTAGCTTTTATTTTGCTCCGCGAAC |
| **B. Primers for *E. coli* colony PCR and *Synechocystis* colony PCR** | |
| T7_SF | GCGAAATTAATACGACTCACTATAG |
| VF2 | TGCCACCTGACGTCTAAGAA |
| VR | ATTACCGCCTTTGAGTGAGC |
| kivd_SR | CGTTGCCAACCCATTTCATATC |

**Reference:**

1. Catanzariti AM, Soboleva TA, Jans DA, Board PG, Baker RT. An efficient system for high-level expression and easy purification of authentic recombinant proteins. *Protein Sci*. 2004;13(5):1331-9.
